# Negative Regulation of Receptor Tyrosine Kinase (RTK) Signaling: A Developing Field

**Published:** 2007-02-14

**Authors:** Fernanda Ledda, Gustavo Paratcha

**Affiliations:** Laboratory of Molecular and Cellular Neuroscience, Department of Neuroscience, Karolinska Institute, Retzius väg 8, 17177, Stockholm, Sweden

**Keywords:** Receptor tyrosine kinases (RTKs), Trophic factors, signal transduction, negative control and cancer

## Abstract

Trophic factors control cellular physiology by activating specific receptor tyrosine kinases (RTKs). While the over activation of RTK signaling pathways is associated with cell growth and cancer, recent findings support the concept that impaired down-regulation or deactivation of RTKs may also be a mechanism involved in tumor formation. Under this perspective, the molecular determinants of RTK signaling inhibition may act as tumor-suppressor genes and have a potential role as tumor markers to monitor and predict disease progression. Here, we review the current understanding of the physiological mechanisms that attenuate RTK signaling and discuss evidence that implicates deregulation of these events in cancer.

## Introduction

The signals that control cell fate determination and coordinate the development of the organs needs to be exquisitely regulated in both time and space. Activation of RTKs by their cognate trophic factors is among the signals that are critically involved in morphogenesis, inducing signaling pathways that control cellular processes such as cell proliferation, differentiation, migration and survival (Ullrich and Schlessinger, 1990). Thus, to avoid signaling errors that ultimately lead to aberrant cellular behavior and disease, cellular mechanisms have evolved to ensure that appropriate signaling thresholds are achieved and maintained during the right period of time.

RTKs are single spanning transmembrane proteins possessing an intrinsic kinase activity. Upon ligand binding, the kinase is activated and autophosphorylates itself on tyrosine residues located within the cytoplasmic tail, creating docking sites for proteins containing phosphotyrosine-binding domains and forming the starting-point for a variety of different signaling cascades that regulate cell physiology. In particular, Ras-Erk/MAP kinase and phosphatidylinositide-3 kinase (PI3K)-AKT pathways represent two critical signaling cascades induced upon the activation of RTKs by trophic factors (Blume-Jensen and Hunter, 2001).

The deregulation of approximately fifty percent (30 of 58) of the genes known to encode RTKs are associated with human tumors (Blume-Jensen and Hunter, 2001). Several mechanisms that increase the catalytic activity of RTKs (positive signaling) have been identified. Examples of these mechanisms include chromosomal translocation, receptor amplification and point mutations (Blume-Jensen and Hunter, 2001; Lamorte and Park, 2001). Since over activation of RTK signaling has been implicated in the onset and progression of different human disorders and cancer, it is essential to understand how RTKs are down-regulated and deactivated. Unlike positive signals, which are relatively well understood, the molecular mediators of signal desensitization are currently under intensive study. During the last years, biochemical and genomic techniques as well as genetic analyses of developmental processes have led to the identification and characterization of the mechanism of action of several RTK signaling inhibitors (Table 1). These studies have underscored the importance of negative-feedback control of RTK function as a mechanism to ensure signaling thresholds compatible with the induction of a physiological response (Casci and Freeman, 1999; Fiorini et al. 2002; Ghiglione et al. 1999; Golembo et al. 1996; Tsang and Dawid, 2004). A common feature of these feedback loops is the transcriptional induction of negative attenuators by the same pathways that are eventually inhibited (late attenuators). Negative feedback is one of the mechanisms that provide an effective control of RTK signaling. However other mechanisms, collectively known as receptor down-regulation, have been evolved to restrict RTK signaling independently of transcription (early attenuators) (Haglund et al. 2003b; Thien and Langdon, 2001). This type of molecular machinery exists prior to receptor activation thereby limiting signal propagation through promoting the receptor ubiquitination, endocytosis and degradation.

**Figure 1:**
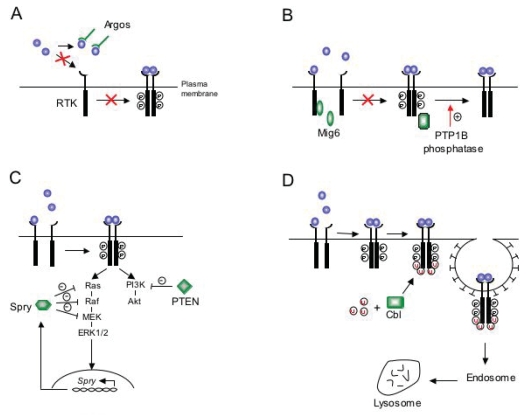
Different mechanisms of RTK signal attenuation. **(A)** Ligand-sequestration and binding inhibition. This panel illustrates the inhibitory role of the secreted protein Argos, which negatively regulates DER signaling sequestering the DER-activating ligand Spitz and preventing Spitz binding to DER. **(B)** Inhibition of RTK autophosphorylation. Examples of this type of inhibition include the cytosolic adapter/scaffold protein Mig6/Ralt/Gene33 and the PTP1B phosphatases. Mig6 binds to the intracellular domain of the EGFR and inhibits its autophosphorylation. Another way by which EGFRs can become deactivated is by the action of PTP1B protein tyrosine phosphatases that reduce ErbB2 receptor phosphorylation. **(C)** Inhibitory proteins that counteract downstream signaling. Trophic factor stimulation activates the Ras-Erk1/2 pathway, which ends in the induction of the *Sprouty* gene. Then, Sprouty in a negative-feedback loop deactivates this cascade by inhibiting the pathway at undetermined intermediates. The role of the phosphatidylinositol phosphatase PTEN as a specific attenuator of the RTK-PI3K-Akt pathway is also indicated. **(D)** Ligand-induced receptor ubiquitination and degradation. This panel illustrates the mechanism of RTK down-regulation mediated by the ubiquitin ligase c-Cbl. Trophic factor binding to a RTK induces receptor autophosphorylation via receptor dimerization, followed by the subsequent activation of the Ras-Erk1/2 and PI3K-Akt signaling pathways. The ubiquitin-ligase c-Cbl interacts with the tyrosine-phosphorylated RTK and mediates its multi-ubiquitination. Receptor ubiquitination facilitates endocytosis and posterior lysosomal degradation of activated RTKs.

RTKs coordinate a wide variety of biological processes and are therefore subjected to multiple levels of control. Multiple modes of action have been described to inhibit RTK signaling. In Figure 1, we illustrate this concept describing the mechanisms through which different physiological inhibitors antagonize and restrict trophic factor signaling, including ligand sequestration and binding inhibition, attenuation of RTK autophosphorylation, induction of inhibitory proteins that counteracts downstream signaling pathways and ligand-induced receptor ubiquitination. Therefore, this review focuses on recent advances made in the understanding of the physiological mechanisms that restrict RTK signaling and summarizes their putative dysfunction in neurological diseases and cancer.

## Mechanisms of RTK Signaling Attenuation

### Ligand sequestration and binding inhibition

In Drosophila, activation of epidermal growth factor receptor (EGFR) homologue, DER is strictly regulated. DER is a receptor tyrosine kinase required for developmental processes throughout life cycle (Perrimon and Perkins, 1997; Schweitzer and Shilo, 1997) and different mechanisms have been described for modulation of this signaling. The secreted protein Argos is the only known extracellular inhibitor of DER (Schweitzer et al. 1995) with a clear physiological role during development. Argos is a secreted protein of 444 aa with an atypical EGF-like motif (Freeman et al. 1992) identified as an inhibitor of DER signaling by genetic deletions (Freeman et al. 1992; Golembo et al. 1996; Wasserman and Freeman, 1998). While Argos mutant embryos show hyperactivation of DER signaling (Golembo et al. 1996), the addition of Argos resulted in the abrogation of DER activation by its ligand, Spitz (Schweitzer et al. 1995), indicating a role to Argos as a negative regulator of DER. Although several reports have argued that Argos interacts directly with DER (Jin et al. 2000; Schweitzer et al. 1995; Vinos and Freeman, 2000), recently it has been shown that Argos inhibits DER signaling by sequestering its activating ligand (Klein et al. 2004). During the last years Kekkon1 emerged as a new inhibitor of DER. In contrast to Argos, Kekkon1 is a single spanning transmembrane protein with leucine-rich repeats (LRR) and immunoglobulin (Ig) motifs (Musacchio and Perrimon, 1996). In developmental assays, it was demonstrated that loss of Kekkon1 activity results in increased DER signaling, whereas ectopic expression of the gene suppressed receptor activation, suggesting that Kekkon acts as a negative regulator of DER activity. In this case, the inhibition involves a physical interaction between both the extracellular and transmembrane domains of Kekkon1 with DER (Ghiglione et al. 1999). Thus, Kekkon1 inhibits ligand binding and autophosphorylation of the receptor, resulting in the suppression of downstream signaling events. Interestingly, the expression of Argos as well as Kekkon1, are induced by DER activation (Ghiglione et al. 1999; Golembo et al. 1996; Schweitzer et al. 1995) therefore representing a negative feedback mechanism.

Although we do not know yet whether a mammalian Argos equivalent exists, the mammalian protein structurally related to Kekkon1, LRIG1 (leucine-rich repeats and immunoglobulin-like domain 1) has been described. Interestingly, LRIG1 has been shown to inhibit mammalian EGFR activation by a different mechanism (see Table 1).

Even though evidences for RTK regulation by ligand sequestration come from studies made in Drosophila, they suggest new possible strategies for the design of novel anti-oncogene agents. Future studies are required to develop new reagents that can neutralize RTK ligands, which overexpression is involved in the development of different malignancies.

During the last years, a new mechanism of RTK negative regulation was described (Andl and Rustgi, 2005). In contrast to ligand sequestration, this mechanism involves a decrease in the RTK ligand affinity, mediated i.e. by E-cadherin. Although E-cadherin was originally described as a structural cell surface glycoprotein involved in cell-cell adhesion, later it was shown to have signaling function. During the last years, E-cadherin was found to interact through its extracellular domain with EGFR, Hepatocyte growth factor receptor (HGFR/MET) and Insulin-like growth factor receptor (IGFR-1), thereby decreasing receptor mobility and its affinity for their ligand. Interestingly, the interaction of E-cadherin with RTKs does not impair E-cadherin dimerization and adhesive function (Qian et al. 2004). Thus, the increased cell motility and invasiveness observed in E-cadherin negative tumors that has been usually attributed to the loss of cell adhesion, could also be explained in part by the loss of cell adhesion-dependent RTK inhibition.

### Inhibition of RTK autophosphorylation

Inhibition of RTK autophosphorylation can be achieved by protein tyrosine phosphatases (PTPs). Several studies have shown that PTPs specifically dephosphorylate certain subsets of phosphorylated tyrosines on RTKs that have multiple phosphorylation sites, indicating a certain degree of selectivity (Kovalenko et al. 2000; Ostman et al. 2006; Persson et al. 2004). Several PTPs has been reported to be able to regulate RTK activity by abrogating receptor autophosphorylation and subsequently blocking downstream signaling. The inappropriate activity of PTPs leads to aberrant tyrosine phosphorylation that contributes to the development of cancer (Hunter, 2000). One example is the phosphatase PTP1B that impairs EGFR activation (Lammers et al. 1993). Fibro-blasts from PTP1B-deficient mice show an increase and sustained EGFR phosphorylation after growth factor treatment (Haj et al. 2003). Another example of RTK-regulating tyrosine phosphatase is the RPTPσ, whose activity has been implicated in the negative regulation of EGF receptor activation and downstream signaling (Suarez Pestana et al. 1999). Recently, the PEST-type protein-tyrosine phospha-tase BDP1 emerged as a new regulator of ErbB2. While the overexpression of BDP1 inhibited ligand-induced activation of ErbB2, the suppression of endogenous BDP1 expression increased its phosphorylation. Moreover, BDP1 was able to interfere with downstream signaling events, reducing MAPK activation (Gensler et al. 2004).

SHP-1 has also been identified as a phosphotyrosine phosphathase that negatively regulates the nerve growth factor (NGF) receptor, TrkA. SHP-1 interacts with TrkA at tyrosine 490 and controls both the basal NGF-stimulated level of TrkA activity in developing peripheral neurons (Marsh et al. 2003). Another potential negative regulator of Trk signaling is the SLAM-associated protein (SAP). SAP protein interacts with the TrkA, TrkB and TrkC neurotrophin factor receptors *in vitro* and *in vivo*. Binding of SAP requires Trk receptor activation and phosphorylation of the tyrosine 674, which is located in the activation loop of the kinase domain. Moreover, overexpression of SAP attenuates tyrosine phosphorylation of Trk receptors and suppress NGF-induced neurite outgrowth (Lo et al. 2005).

During the last years Mitogen-inducible gene 6 (Mig6 also known as RALT or Gene 33) was identified as a feedback inhibitor of different RTKs. Several studies indicate that Mig6 can attenuate mitogen signaling induced by EGF, Heregulin (HRG-β) and Hepatocyte growth factor (HGF/ MET) (Fiorentino et al. 2000; Fiorini et al. 2002; Hackel et al. 2001; Pante et al. 2005; Xu et al. 2005). The molecular mechanisms underlying the inhibition achieved by Mig6 is still controversial. In the case of EGF and ErbB2 receptors, it was shown that Mig6 is able to suppress their signaling by directly binding to the RTKs and inhibiting the EGFR/ErB2 receptor autophosphorylation therefore attenuating the MAPK signaling (Anastasi et al. 2003). In line with these results, the deletion of the mouse gene encoding Mig6 shows hyperactivation of endogenous epidermal growth factor receptor and sustained signaling through MAPK, resulting in the overproliferation and impaired differentiation of keratinocytes (Ferby et al. 2006). Moreover, these mice develop spontaneous tumors in different organs supporting a role for Mig6 as a novel tumor suppressor of EGFR-dependent malignancies. On the other hand, Mig6 was found to inhibit the signaling triggered by HGF by indirectly binding to its tyrosine kinase receptor, MET, through the adaptor protein Grb2 (Pante et al. 2005). Interestingly, it was shown that part of Mig6’s mechanism of action involves the inhibition of the GTPase Cdc42. The overexpression of Mig6 was able to inhibit the HGF/MET-induced cell migration and neurite outgrowth (Pante et al. 2005). However, the physiological relevance of MET attenuation in Mig6-deficient mice has not been reported and deserves additional analysis.

Few natural ligands that inhibit RTK activation have been identified to date. Herstatin protein belongs to this short list of natural ligands that attenuate RTKs. Herstatin is a secreted product of the *ErbB2* gene containing a truncated extracellular domain. Herstatin has been shown to disrupt receptor dimerization and reduce ErbB2 receptor phosphorylation (Doherty et al. 1999) More recently, Hu et al. (2006) reported a novel and intracellular mechanism by which Herstatin could attenuate ErbB2 receptor activity. In this case, Herstatin has the ability to reduce ErbB2 receptor levels on the cell surface by sequestration of ErbB2 receptors in the endoplasmic reticulum (ER). In this model, Herstatin decreases ErbB2 receptor translocation from ER to cell surface (Basson et al. 2005; Hu et al. 2006).

### Inhibitory Proteins that Counteract Downstream Signaling

The majority of the biological processes induced upon RTK engagement require the precise stimulation of Erk/MAP kinase family members and activation of PI3K and Akt kinases. Increasing interest in negative regulation of RTK signaling has led to the identification of different pathway-specific inhibitors. Although during the last years several negative regulators of RTK downstream signaling have been described, mounting evidence highlights the role of Sprouty, Sef and PTEN proteins as both selective and physiological inhibitors of Erk/MAPK and PI3K-Akt signaling pathways respectively.

The Sprouty (Spry) family of proteins has emerged as a major class of trophic factor-inducible antagonists of RTK signaling. In particular, Sprouty proteins appear to specifically inhibit the Ras-Raf-Erk1/2 pathway, leaving the PI3K and other MAPK pathways intact (Gross et al. 2001; Yusoff et al. 2002). The negatively regulated mammalian RTKs include Fibroblast growth factor receptor (FGFR), Hepatocyte growth factor receptor (HGFR/MET), Vascular endothelial growth factor receptor (VEGFR) and Glial cell-line derived neurotrophic factor (GDNF) receptor, RET (Impagnatiello et al. 2001; Kramer et al. 1999; Reich et al. 1999; Sasaki et al. 2003). The levels at which Sprouty proteins block Erk/MAPK activation are still unclear and the evidence to date suggest the existence of mechanisms that depend on the cellular context and the RTK considered.

More recent biochemical and genetic evidence indicate specific roles for the *Sprouty* genes during normal development and multiple modes of action of the Sprouty proteins in the regulation of RTK-induced responses. As a negative regulator, Sprouty itself is subject to tight control at multiple levels. Specifically, growth factors increase the levels of the *Sprouty* transcripts, regulate the recruitment of Sprouty proteins to the plasma membrane and modulate Sprouty activity through rapid and transient tyrosine phosphorylation (Y55) (Mason et al. 2004). In particular, phosphorylation of Sprouty proteins on a tyrosine residue located at position 55 is required for its ability to inhibit RTK-induced Ras-Erk1/2 signaling (Mason et al. 2004; Sasaki et al. 2001) However, phosphorylation of this evolutionarily conserved tyrosine is also necessary for the interaction of Sprouty with c-Cbl, an E3 Ubiquitin ligase that mediates the direct ubiquitination and degradation of several RTKs (Hall et al. 2003; Mason et al. 2004; Rubin et al. 2003). Therefore, Sprouty protein levels are controlled through a phosphorylation-dependent complex formed with c-Cbl. Polyubiquitination and degradation of an active Sprouty might limit its inhibitory effects to a defined period after receptor engagement. Intriguingly, several studies have also demonstrated that mammalian Sprouty proteins can increase EGF-mediated Erk/MAPK signaling in a cell type-dependent manner (Egan et al. 2002; Rubin et al. 2003; Wong et al. 2002). This novel agonistic effect of Sprouty is strictly dependent on c-Cbl. In this particular case, Sprouty bound to c-Cbl, competes and prevents c-Cbl-mediated ubiquitination and down-regulation of activated EGF receptors (EGFRs), yielding sustained levels of activated EGFR and resulting in a net increase in downstream signaling. In summary, the c-Cbl-Sprouty interaction emerges as a critical signaling event important in controlling the antagonistic function of Sprouty and, at the same time, the life cycle of Sprouty proteins themselves.

Another molecule that belongs to this category of inhibitors is Sef (Similar expression to *fgf* genes). This newly identified antagonist encodes a putative Type I transmembrane protein that is conserved across zebrafish, mouse and human (Kovalenko et al. 2003). Sef protein restricts FGFR signaling by acting as a feedback-induced antagonist of the Ras/MAPK-mediated FGF signaling (Furthauer et al. 2002). Interestingly, mouse Sef (mSef) also attenuates FGF-induced activation of PKB (PKB/AKT), a key protein in the PI3K pathway (Kovalenko et al. 2003). Neverthless, the precise mechanism of the inhibitory effect of Sef remains controversial, since it has also been reported that Sef may antagonize FGF signaling by binding to and restricting FGFR tyrosine phosphorylation (Kovalenko et al. 2003). In addition, it has been reported that alternative splicing of the human *Sef* (*hSef*) gene alter the subcellular localization of this protein and diversify the repertoire of RTKs to be inhibited (Preger et al. 2004).

Finally, PTEN (also referred to as MMAC1 and TEP1) is another attenuator that has been implicated in negative signaling by RTKs. This phosphatidylinositol phosphatase is implicated in negative signaling that specifically inhibits PI3K-Akt signaling pathway triggered by RTKs. This pathway is a key regulator of cell proliferation, motility and survival. The activity of Akt is regulated by PI3K via the synthesis of phosphatidyl inositol 3, 4, 5-triphosphate (PIP3). PTEN antagonizes PI3K by degrading PIP3 to phosphatidyl inositol 4, 5-biphosphate (PIP2). Deregulation of Akt through loss of functional PTEN has been implicated in the progression of different tumors (Simpson and Parsons, 2001). In agreement with this, the down-regulation of PTEN results in an increased concentration of PIP3 and Akt hyperactivation leading to protection from apoptotic stimuli (Stambolic et al. 1998). In contrast, over-expression of PTEN in cancer cell lines results in the inactivation of Akt and cell cycle arrest (Lu et al. 1999).

### Ligand-induced Receptor Ubiquitination and Degradation (Receptor Down-regulation)

Down-regulation of RTKs is an irreversible mechanism of inhibition that regulates the extent of the signal by removing activated receptors from the plasma membrane. Once activated, RTKs are ubiquitinated, internalized and targeted for degradation to the lysosomal compartment or locally destroyed in proteasomes. Many of these processes are regulated by ubiquitination, a post-traslational modification where the small protein ubiquitin is covalently attached to a target protein. While poly-ubiquitination marks proteins for proteasomal degradation, mono- or multi-ubiquitination is sufficient to direct endocytosis and lysosomal degradation of membrane receptors (Haglund et al. 2003a; Haglund et al. 2003b; Thien and Langdon, 2001).

Central to the process of receptor down-regulation are the roles of Cbl and Nedd families of ubiquitin-protein ligases, which act through limiting signal propagation independently of new transcriptional events (Harvey and Kumar, 1999; Thien and Langdon, 2005). Recently, Arevalo et al. (2006) have identified the E3 Ubiquitin ligase Nedd4-2 as an enzyme that binds specifically to the c-terminal portion of the TrkA receptor (Arevalo et al. 2006). The binding of Nedd4-2 to activated TrkA leads to the ubiquitination and down-regulation of TrkA and to the modulation of neuronal survival by NGF. In contrast, several other activated receptors, such as EGFR (ErbB1), Platelet-derived growth factor receptor (PDGFR), RET and MET receptors are ubiquitinated upon interaction with c-Cbl, the most studied member of the Cbl family. By virtue of their tyrosine kinase-binding (TKB) domain, c-Cbl can directly associate with activated receptors by the binding of its SH2 domain to specific tyrosine residues on the receptor. However, Cbl also interacts with the SH3 domain of Grb2, an adaptor protein known to associate with phosphorylated receptors and link RTK to the activation of the Ras pathway. Thus, in mammalian cells Grb2 can indirectly recruit c-Cbl to EGFR, MET and RET receptors (Jiang et al. 2003; Scott et al. 2005).

Consistent with its role in the down-regulation of RTKs, dominant negative mutants of c-Cbl lacking ubiquitin ligase activity have been identified in mouse tumors (Thien and Langdon, 2001). In addition to the targeting of RTKs for lysosomal degradation after ubiquitination, several evidences support a role for c-Cbl in the endocytosis of RTKs. In particular, the overexpression of c-Cbl increases the rate of EGFR internalization (Soubeyran et al. 2002). It has been shown that c-Cbl promotes the internalization of RTKs by binding to the CIN-85-Endophilin complex, a step required for the invagination of the plasma membrane into the coated-pits (Petrelli et al. 2002; Soubeyran et al. 2002). Inhibition of Cbl-CIN85-Endophilin interaction was sufficient to block RTK endocytosis and degradation, without disrupting the ability of Cbl to ubiquitinate activated receptors (Petrelli et al. 2002; Soubeyran et al. 2002).

Recent studies have also linked RTK ubiquitination to receptor endocytosis (Marmor and Yarden, 2004; Mosesson et al. 2003). A number of endocytic regulatory proteins have been demonstrated to interact with ubiquitin and coordinate the trafficking of ubiquitinated RTKs from endosomes to lysosomes. Examples include Hrs, Eps15, Stam, Epsin and Tsg101 proteins. Interestingly, the kinetic properties and the magnitude of the signaling response of the RTK may be regulated by the location of the activated receptor along the endocytic pathway (Burke et al. 2001). Since RTKs can transmit signals from the membrane of the endosomes, the molecular machinery that control the trafficking of receptors from early endosomes to degradative lysosomes represent key proteins in the down-regulation of receptor signals. Therefore, any alterations that uncouple RTKs from ligase-mediated ubiquitination, internalization and down-regulation are tightly associated with cancer.

Recent studies have identified the mammalian leucine-rich repeats and immunoglobulin-like domain 1 (LRIG1) protein as an endogenous inter-actor of c-Cbl (Gur et al. 2004; Laederich et al. 2004). LRIG1 is a transmembrane protein with an ectodomain containing 15 leucine-rich repeats (LRRs) and three immunoglobulin-like motifs. The structural similarity of LRIG1 with other inhibitors previously described in insects (Kekkon) led to the prediction that LRIG1 could interact and restrict EGF signaling in mammalian cells. Notably, disruption of the *Lrig1* gene in mice resulted in fertile animals that develop skin defects, suggesting involvement in EGFR signaling regulation (Suzuki et al. 2002). Based on these two evidences, it has been reported that LRIG1 is a negative feedback regulator of the four EGFR mammal orthologs (ErbB1, ErbB2, ErbB3 and ErbB4). The underlying mechanism involves the upregulation of LRIG1 and a subsequent direct EGFR-LRIG1 interaction followed by an enhanced recruitment of c-Cbl leading to accelerated ubiquitination and degradation of EGFRs (Gur et al. 2004; Laederich et al. 2004).

Based on the premise that the LRR domain is the critical interacting domain between LRIG1 and the EGF/ErbB receptor family, Goldoni et al. (2006) demonstrated that a soluble ectodomain of LRIG1, containing only the LRRs, repress both ligand-independent and ligand-dependent EGFR activation and ERK1/2 signaling in a dose-dependent manner (Goldoni et al. 2006). In contrast to the entire protein, this attenuation occurs without any significant internalization and degradation of the receptor. Thus, inhibition of EGFR activity without down-regulation of the receptor could represent a novel therapeutic approach toward malignancies in which EGFR has a primary role promoting tumor growth.

Finally, Decorin represents another example of the negative regulation of EGF/ErbB receptors by proteins containing leucine-rich repeats. Decorin is a secreted proteoglycan molecule that acts as an inhibitor of mammalian EGFRs (Iozzo et al. 1999). In particular, Decorin leads to the protracted internalization and degradation of the EGFR (Zhu et al. 2005). Additional inhibition of EGF-mediated EGFR dimerization and activation by soluble Decorin has been reported. Interestingly, this novel and dual mechanism of action could explain the anti-oncogenic properties of Decorin.

## Repressors of RTK Signaling as Tumor-suppressor Genes

Over activation of RTK signaling is a common feature of cellular transformation and malignancy. Based in this concept, several groups began investigating the role of negative regulators of trophic factor-mediated signaling in cancer. They described that the expression of specific RTK attenuators is down-regulated in different types of human cancer. These studies led to the identification of specific tumor-suppressor genes, whose dysfunction or their down-regulation results in promoting malignancy. In this section, we highlight recent progresses in understanding the defective attenuation of RTK signaling in cancer and discuss their potential for the development of effective therapeutic approaches. In particular, we provide evidences for a role of the protein attenuators: PTEN, Mig6, LRIG1 and Sprouty in human cancer.

## PTEN

The tumor suppressor PTEN is localized at chromosome 10q23, which has been observed to be mutated in different sporadic cancers. Loss of chromosome 10q is the most common genetic alteration that is associated with the most aggressive form of glioma, glioblastoma multiforme (GBM) (Fults and Pedone, 1993; von Deimling et al. 1993). Several reports have indicated a high frequency of PTEN mutations in glioblastomas (Chiariello et al. 1998; Duerr et al. 1998; Liu and Chernoff, 1997; Teng et al. 1997; Wang et al. 1997). Studies at the level of PTEN expression in glioblastomas versus lower grade gliomas suggest that the reduction of PTEN is important in the progression from gliomas to GBM stage (Sano et al. 1999). Another tumor type that frequently exhibits loss of chromosome 10q is prostate carcinoma. PTEN mutations have been described in this carcinoma and it has been suggested that the inactivation of PTEN occurs mainly in advanced prostate cancer. In agreement with this, PTEN immunohistochemical analysis has correlated its decreased expression with pathological markers of poor prognosis (McMenamin et al. 1999). Thus, down-regulation of PTEN expression may play a role in the development of advanced prostate cancer. Interestingly, homozygous inactivation of *Pten* is embryonic lethal in mice. However, *Pten* ^+/−^ mice show hyperplastic-dysplastic features and are highly susceptible to develop epithelial tumors (Di Cristofano et al. 1998; Di Cristofano et al. 2001; Podsypanina et al. 1999; Suzuki et al. 1998). Recently, it has been described that conditional prostate-specific deletion of the murine *Pten* gene leads to metastatic prostate cancer (Wang et al. 2003). In melanoma, PTEN mutations also appear to be associated with late stages of the disease (Reifenberger et al. 2000; Tsao et al. 2000; Zhou et al. 2000). Different studies reported that the frequency of PTEN mutations is higher in patients with metastatic melanoma compared with primary tumors (Reifenberger et al. 2000; Tsao et al. 2000). Several studies examining endometrial carcinomas indicate a high frequency (approximately 50%) of PTEN mutations (Risinger et al. 1997; Tashiro et al. 1997). Interestingly, only a small fraction of breast cancer cases, show mutations in PTEN (Bose et al. 1998; Feilotter et al. 1999; Rhei et al. 1997; Ueda et al. 1998).

It is known that the recombinant anti-ErbB2 monoclonal antibody, Herceptin, has remarkable therapeutic efficacy in patients with ErbB2-overexpressing tumors. The mechanism underlying Herceptin’s antitumor activity includes the down-regulation of p185^ErbB2^ receptor and the subsequent inhibition of its downstream PI3K-Akt signaling pathway (Hudziak et al. 1989; Yakes et al. 2002). Despite this, the causes of Herceptin resistance are not well understood. Recently, it has been reported that loss of PTEN in breast cancer cells overex-pressing ErbB2 confers resistance to Herceptin treatment (Nagata et al. 2004). In particular, patients with PTEN-deficient breast cancers have significantly poorer responses to Herceptin-based therapy than those with normal PTEN, suggesting that PI3K-targeting therapies could overcome this resistance.

## Mig6

Mig6 chromosomal locus is located within the region 1p36.1–3, which has been described to be mutated in different human cancers (Koshikawa et al. 2004; Ogunbiyi et al. 1997; Tseng et al. 2005). During the last years it has been reported that Mig6 expression is down-regulated in patients with breast cancer and short survival time (Amatschek et al. 2004). More recently, it was shown that Mig6 expression is reduced in skin, breast, pancreatic and ovarian carcinomas (Ferby et al. 2006). Interestingly the loss of Mig6 in mice results in the hyperactivation of endogenous EGFR signaling, a high incidence of neoplastic lesions, and high susceptibility to carcinogen-induced formation of papillomas and melanomas (Ferby et al. 2006). These findings suggest that loss of Mig6 may be used as a novel marker in the process toward malignancy.

## LRIG1

LRIG1 is located at chromosome 3p14.3, which has been reported to be mutated in different tumor types. Its expression is down-regulated in tumor cell lines derived from lung, prostate and colon when compared to normal tissue (Hedman et al. 2002). Loss of heterozygosity at the LRIG1 locus was found in human breast cancers (Maitra et al. 2001). Another malignancy where LRIG1 expression was found to be down-regulated is renal cell carcinoma (Thomasson et al. 2003). Moreover, in squamous cell carcinoma, low levels of LRIG1 expression has been correlated with increased metastasis and poor patient survival, (Tanemura et al. 2005) suggesting that the down-regulation of LRIG1 provides a novel prognostic predictor in this malignancy.

## Sprouty

Down-regulation of Spry1 and Spry2 has been described in breast cancer. Using cDNA arrays containing pairs of cDNAs generated from tumor and normal tissue samples from individual patients, a high frequency in the down-regulation of Spry1 (78%) and Spry2 (96%) have been observed. These findings were confirmed by real time PCR (Lo et al. 2004). Different studies indicate that Sprys are also down-regulated in prostate cancer. Using tissue microarrays containing pairs of samples from tumors and normal peripheral tissue, Spry1 was shown to be decreased in 39% of prostate cancer compared with matched normal prostate tissue (Kwabi-Addo et al. 2004). However, a considerable fraction of the tumors exhibited a higher expression of Spry1 to the corresponding peripheral tissue indicating that, although decreased Spry1 expression is seen in a substantial fraction of prostate cancers, loss of Spry1 expression is not required in all prostate cancers. In a more recent study, it was reported that Spry2 expression is reduced in high-grade clinical prostate cancer when compared to benign prostatic hyperplasia (McKie et al. 2005). Studies in renal cell carcinomas indicate that Spry1 is upregulated in patients with a good outcome. More recently it was described that the expression of Spry2, but not Spry1, is down-regulated in liver cancer (Fong et al. 2006). The mechanisms by which Spry is down-regulated in the different cancer types remain unclear but might be specific to different malignancies. Although in the cases presented above, Spry expression seems to be a marker for good clinical prognosis, in other cancer types Sprouty expression is controversial. Tsavachidou et al. (2004) has described an upregulation of Spry2 in melanoma cells with B-Raf V599E mutations compared to melanocytes with wild type B-Raf (Bloethner et al. 2005; Tsavachidou et al. 2004). Therefore, future studies are needed to define whether Spry has a tumor suppressor role.

## Conclusions and Perspectives

Recent advances in the understanding of the mechanisms involved in down-regulating RTKs and restricting their signaling has been summarized above. The overall picture that emerges from these studies indicates that the mechanisms of regulation occur at numerous levels, including ligand binding, receptor autophosphorylation, induction of inhibitory proteins that counteract downstream signaling pathways and receptor endocytosis and degradation. Another important concept is related to the fact that negative feedback loop is one of the mechanisms that has evolved to provide an effective way of controlling RTK-mediated signaling.

Conclusions derived from the work outlined in this review indicate that RTK activity is tightly controlled through the coordinated action of several negative protein regulators that function at multiple levels of the signaling cascade, and at different time-points after receptor engagement. Recent evidence also demonstrates that certain inhibitors have multiple mechanisms of action that depend on the cellular context and the identity of the RTK inhibited.

In addition to protein attenuators, microRNAs (miRNAs) have emerged as an abundant class of small (approx. 22-nucleotides) non-protein-coding RNAs that play an important role in the negative regulation of gene expression, controlling the translational efficiency of target mRNAs (Esquela-Kerscher and Slack, 2006). MicroRNAs have been shown to regulate a wide range of developmental processes modulated by RTKs, like proliferation, survival and differentiation. Recently, several miRNAs have been associated with human cancer. Interestingly, miRNAs can function as tumor-suppressor and oncogenes (Lee and Dutta, 2006; Mendell, 2005) and might become a powerful tool to aid in the diagnosis and treatment of cancer. Despite the advances in the identification of specific miRNAs, our understanding of their target mRNAs in normal and pathological conditions is at very preliminary stage. Therefore, future studies will help to elucidate whether miRNAs could represent a new and alternative mechanism to down-regulate RTK signaling during normal development and disease.

Many important aspects of RTK signaling inhibition still remain unanswered. One significant issue that requires a more detailed investigation is the exact identification of the signaling pathways regulated by these inhibitory molecules and, particularly, their *in vivo* function.

The elucidation of the mechanisms that control RTK activation is today seen as one of the major challenges in biomedical science. Neurotrophic factor signaling through their RTK receptors play critical roles in the development of the nervous system, in the survival and maintenance of specific subpopulations of differentiated neurons. While the over activation of RTK signaling due to impaired deactivation of RTK signaling is associated with cancer, it is also possible that alterations in these control mechanisms could contribute to the pathogenesis of neurodevelopmental diseases and neuro-degenerative disorders. In particular, individuals with germline PTEN mutations display brain disorders including macrocephaly, seizures and mental retardation (Waite and Eng, 2002). Interestingly, PTEN mutations has been reported in individuals with autism spectrum disorders (ASD) (Butler et al. 2005; Goffin et al. 2001; Zori et al. 1998). A recent study showed that deletions of *Pten* in the mouse central nervous system can underlie macrocephaly and behavioral abnormalities reminiscent of certain features of human ASD (Kwon et al. 2006). Therefore, another general issue consists in identify and describe the expression of these attenuators in tissue isolated from different neurological pathologies.

Finally, a more complete understanding of these emergent mechanisms will have wide implications in medicine, particularly in the identification of tumor-suppressor markers and in the design of efficient therapeutic approaches to human diseases.

## Figures and Tables

**Table 1:** Classification of RTK signaling inhibitors according to their mechanisms of action.

Attenuator	Type of attenuator	Inhibitory target	Mechanism of action	References
Argos	Late/reversible	Drosophila EGFR (DER)	Ligand sequestration	Klein et al. 2004
Kekkon	Late/reversible	DER	Inhibition of trophic factor binding	Ghiglione et al. 2003
E-Cadherin	Early/reversible	EGFR, IGFR and Met receptor	Adhesion-dependent RTK inhibition. Decrease of ligand affinity	Qian et al. 2004
BDP1 phosphatase	Early/reversible	ErbB2R	Reduction of ErbB2R autophosphorylation	Gensler et al. 2004
Herstatin	Early/reversible	ErbB2R	Reduction of ErbB2R dimerization and activation. Sequestration of ErbB2R in the ER.	Doherty et al. 2001; Hu et al. 2006
Mig6/Ralt/Gene33	Late/reversible	EGFR, ErbB2 and Met receptors	Inhibition of EGFR/ErbB2R autophosphorylation and Met-Rho-like GTPase pathway	Hackel et al. 2001; Pante et al. 2005
PTP1B phosphatase	Early/reversible	EGFR and IGFR	Reduction of EGFR and IGFR autophosphorylation	Liu and Chernoff 1997; Elchebly et al. 1999
SAP (Slam-associated protein)	Early/reversible	TrkA, TrkB and TrkC	Reduction of Trk receptor autophosphorylation	Lo et al. 2005
Decorin	Early/irreversible	EGFR and ErbBR family members	Inhibition of EGF-dependent EGFR dimerization and induction of protracted internalization and degradation of the EGFR	Iozzo et al. 1999; Zhu et al. 2005
PTEN	Early/reversible	Several RTKs	Inhibition of PI3K-Akt pathway	Stambolic et al. 1998; Lu et al. 1999
Sef	Late/reversible	FGFR	Inhibition of Ras-MAPK pathway	Tsang et al. 2004; Torii et al. 2004
Sprouty	Late/reversible	Several RTKs	Inhibition of Ras-MAPK pathway	Gross et al. 2001; Yusoff et al. 2002
Synaptojanin	Early/reversible	EGFR	Inhibition of PI3K-Akt pathway	Woscholski et al. 1997
c-Cbl	Early/irreversible	Several RTKs	Receptor ubiquitination and degradation	Thien and Langdon 2005
LRIG1	Late/irreversible	EGFR/ErbB receptor family	Enhancement of receptor ubiquitination and degradation	Gur et al. 2004; Laederich et al. 2004
Nedd proteins	Early/irreversible	IGF1R, VEGFR and TrkA	Induction of receptor ubiquitination and down-regulation	Murdaca et al. 2004; Vecchione et al. 2003; Arevalo et al. 2006
Nrdp1	Early/irreversible	ErbB2R, ErbB3R, ErbB4R	Ligand-independent ErbB receptor degradation	Qiu and Goldberg 2002

## Abbreviations

BDP1: Brain-derived phosphatase 1
Cbl: Casitas B-lineage lymphoma
CIN-85: Cbl-interacting protein of 85 kDa
DER: Drosophila EGFR
EGFR: Epidermal growth factor receptor
ERK 1/2: Extracellular signal-regulated kinase 1/2
Grb2: Growth factor receptor-bound protein 2
HER2: Human epidermal growth factor receptor 2
LRIG: Leucine-rich repeats and immunoglobulin-like domain 1
MAPK: Mitogen-activated protein kinase
Mig 6: Mitogen-inducible gene 6
PTEN: Phosphatase and tensin homologue
RET: Rearranged in transformation
RTK: Receptor tyrosine kinase
SH2 domain: Src-homology 2 domain
SH3 domain: Src-homology 3 domain
Spry: Sprouty
